# *International Journal of Molecular Science* Best Paper Award 2013

**DOI:** 10.3390/ijms14024372

**Published:** 2013-02-22

**Authors:** Ophelia Han

**Affiliations:** MDPI AG, Postfach, CH-4057 Basel, Switzerland and MDPI Branch Office, Beijing 101101, China; E-Mail: ophelia.han@mdpi.com; Tel.: +86-10-8152-1170

Since 2012, *International Journal of Molecular Science* has instituted an annual award to recognize outstanding papers in the area of chemistry, molecular physics and molecular biology that meet the aims, scope and high standards of this journal [1].

We are pleased to announce the second “*International Journal of Molecular Science* Best Paper Award” for 2013. Nominations were made by the Section Editor-in-Chiefs of *International Journal of Molecular Science*, with all papers published in 2009 eligible for consideration. The awards are issued for reviews and articles separately. We are pleased to announce that the following five papers were awarded:

## Article Award

First Prize

**Gerard Giraud, Holger Schulze, Till T. Bachmann, Colin J. Campbell, Andrew R. Mount, Peter Ghazal, Mizanur R. Khondoker, Alan J. Ross, Stuart W.J. Ember, Ilenia Ciani, Chaker Tlili, Anthony J. Walton, Jonathan G. Terry and Jason Crain**

Fluorescence Lifetime Imaging of Quantum Dot Labeled DNA Microarrays

*Int. J. Mol. Sci.***2009**, *10*, 1930-1941; doi:10.3390/ijms10041930

Available online: http://www.mdpi.com/1422-0067/10/4/1930

Second Prize

**Johannes Ranke, Alaa Othman, Ping Fan and Anja Müller**

Explaining Ionic Liquid Water Solubility in Terms of Cation and Anion Hydrophobicity

*Int. J. Mol. Sci.***2009**, *10*, 1271-1289; doi:10.3390/ijms10031271

Available online: http://www.mdpi.com/1422-0067/10/3/1271

Third Prize

**Senthil Chinnasamy, Balasubramanian Ramakrishnan, Ashish Bhatnagar and Keshav C. Das**

Biomass Production Potential of a Wastewater Alga Chlorella vulgaris ARC 1 under Elevated Levels of CO2 and Temperature

*Int. J. Mol. Sci.***2009**, *10*, 518-532; doi:10.3390/ijms10020518

Available online: http://www.mdpi.com/1422-0067/10/2/518

## Review Award

First Prize

**Ronald D. Hills Jr. and Charles L. Brooks III**

Insights from Coarse-Grained Gō Models for Protein Folding and Dynamics

*Int. J. Mol. Sci.***2009**, *10*, 889-905; doi:10.3390/ijms10030889

Available online: http://www.mdpi.com/1422-0067/10/3/889

Second Prize

**Anna Brückner, Cécile Polge, Nicolas Lentze, Daniel Auerbach and Uwe Schlattner**

Yeast Two-Hybrid, a Powerful Tool for Systems Biology

*Int. J. Mol. Sci.***2009**, *10*, 2763-2788; doi:10.3390/ijms10062763

Available online: http://www.mdpi.com/1422-0067/10/6/2763

We believe that these five exceptional papers are valuable contributions to the *International Journal of Molecular Science* and the scientific research field. On behalf of the Prize Awarding Committee and the Editorial Board of *International Journal of Molecular Science*, we would like to congratulate these five teams for their excellent work. In recognition of their accomplishment, Dr. Gerard Giraud, Dr. Johannes Ranke and Dr. Senthil Chinnasamy will receive prizes of 1000 CHF, 800 CHF, and CHF 500, respectively, and the privilege of publishing an additional paper free of charge in Open Access format in the *International Journal of Molecular Science*, after the usual peer-review procedure. Dr. Charles L. Brooks III and Dr. Anna Brückner will receive the privilege of publishing an additional research article free of charge in open access format in the *International Journal of Molecular Science*, after the usual peer-review procedure.

Prize Awarding Committee

*Editor-in-Chief*, Section ‘Material Sciences and Nanotechnology’

**Prof. Dr. Andreas Taubert**

Institute of Chemistry, University of Potsdam, Building 26, Rm. 2.64, Karl-Liebknecht-Str. 24-25, D-14476 Golm, Germany

E-Mail: ataubert@uni-potsdam.de

*Editor-in-Chief*, Section ‘Bioactives and Nutraceuticals’

**Prof. Dr. Charles Brennan**

Department of Food and Tourism Management, Manchester Metropolitan University, Hollings Faculty, Old Hall Lane, Manchester, M14 6HR, UK

E-Mail: charlesbrennan1@yahoo.co.uk

*Editor-in-Chief*, Section ‘Molecular Pathology’

**Prof. Dr. Kurt A. Jellinger**

Institute of Clinical Neurobiology, Kenyongasse 18, A-1070 Vienna, Austria

E-Mail: kurt.jellinger@univie.ac.at

*Editor-in-Chief*, Section ‘Biochemistry, Molecular Biology and Biophysics’

**Prof. Dr. Mark L. Richter**

Molecular Biosciences, Haworth Hall, Room 4031, 1200 Sunnyside Avenue, Lawrence, KS 66045-7534, USA

E-Mail: richter@ku.edu

*Editor-in-Chief*, Section ‘Molecular Diagnostics’

**Prof. Dr. Stephen A. Bustin**

Centre for Academic Surgery, Barts and the London School of Medicine and Dentistry, Queen Mary University of London, 3rd Floor, Alexandra Wing, Royal London Hospital, London E1 1BB, UK E-Mail: stephen.bustin@anglia.ac.uk
